# Prescribers' perspectives: The impact of the controlled substance scheduling system on providing optimal patient care

**DOI:** 10.1016/j.rcsop.2024.100511

**Published:** 2024-09-21

**Authors:** Michael R. Barnes, Yijia Luo, Jonathon M. Parker, Brian M. Shepler

**Affiliations:** a College of Pharmacy, Purdue University, 575 Stadium Mall Dr, West Lafayette, IN 47907, USA; b Cerevel Therapeutics, LLC, 222 Jacobs St Suite 200, Cambridge, MA 02141, USA; c Ultragenyx Pharmaceuticals, 150 Presidential Way, Woburn, MA 01801, USA

**Keywords:** Controlled substances, Prescribing, Healthcare policy, Survey, Research, Pharmacist

## Abstract

**Background:**

In the United States, the scheduling system for controlled substances was established by the Controlled Substance Act of 1970. In 2009, Parker et al. published the study “Physicians' knowledge and attitudes toward scheduling.” Since 2009, the opioid epidemic has gathered national attention from social and scientific perspectives as the number of drug overdose deaths in the United States has nearly tripled.

**Objective:**

To follow up on a 2009 survey by Parker, et al. to determine prescribers' knowledge and attitudes regarding the controlled substance scheduling system and assess the impact of the controlled substance scheduling system on providing optimal patient care.

**Methods:**

The cross-sectional survey was designed to assess prescribers' attitudes and mailed to 400 randomly selected physicians and 400 randomly selected nurse practitioners.

**Results:**

Prescribers across all groups provided consistent responses suggesting an overall lack of understanding of controlled substance regulations, a negative attitude towards the controlled substance scheduling system, and a detrimental effect on providing optimal patient care. Responses from nurse practitioners differed significantly from physicians in 75 % (3 of the 4) questions regarding regulations, suggesting nurse practitioners possess a greater understanding of pharmaceutical regulations. Specialists' responses demonstrated an enhanced level of dissatisfaction regarding the controlled substance scheduling system compared to primary care providers in 75 % (3 of the 4) questions. Questions regarding the impact of the scheduling system on prescribing patterns differed significantly across multiple demographic groups, notably between physicians and nurse practitioners, differences in practice setting, and primary state of practice for 75 % (3 of the 4) questions.

**Conclusions:**

The results of this survey confirm the findings of Parker, et al., and further display the need for investigation into how to improve the controlled substance scheduling system in the United States.

## 1 Introduction

There has been limited published research regarding the utility of the controlled substance scheduling system following the 2009 study “Physicians' knowledge and attitudes toward scheduling” by Parker, et al. surveyed physicians' attitudes regarding the controlled substance scheduling system.^1^ Since its publication in 2009, the opioid epidemic has gathered national attention from social and scientific perspectives as the number of drug overdose deaths in the United States (US) has nearly tripled from 37,000 per year in 2009 to 110,000 per year in 2022.^2^^,^^3^ Given the increased public awareness of the dangers of controlled substance abuse, this research was performed, with modifications, to follow up on the findings from the 2009 study by Parker, et al. and determine if prescribers' views have changed in the decade since that research was conducted.^1^ Based on this increased emphasis on controlled substance abuse, it was anticipated that prescribers' knowledge would have improved in the decade since this previous research was conducted while their attitudes towards the controlled substance scheduling system may have decayed.

In the US, the scheduling system for controlled substances (both prescription pharmaceuticals and illicit substances) was established by the Controlled Substance Act of 1970 (CSA).^4^ During development, prospective medicines under clinical investigation that may have abuse potential are subject to an abuse liability assessment by the pharmaceutical company developing the product, henceforth referred to as the sponsor. If the sponsor submits a New Drug Application, three agencies - United States Food and Drug Administration (FDA), National Institutes of Health and the Drug Enforcement Administration – will collaborate to assess and determine if a medication should be scheduled as a controlled substance utilizing an ‘8-factor analysis’.^5^ Based on this assessment of abuse potential and public health risks, compounds that are designated as a controlled substance are then placed into one of five schedules or categories (Schedule I (CI) through Schedule V (CV)) depending on several factors, including physical and physiological dependence and appropriate medical use.^6^

A controlled substance is defined as medication with the potential for abuse. Schedule I (CI) drugs include compounds such as heroin and are considered to have the highest potential for abuse with no accepted medical use and may not be prescribed, dispensed, or administered within the US.^4^ Starting at CII, these medications have an accepted medical use and as you descend the controlled substance scheduling system, the relative potential for abuse decreases. Examples of controlled substances include opioids in CII, anabolic steroids in CIII, benzodiazepines and anxiolytics in CIV, and antiepileptics (e.g., pregabalin, lacosamide) in CV.^4^ The controlled substance scheduling system is currently intended to inform prescribers of risks associated with addiction, abuse, and misuse of controlled substances, while the numerical aspect of the scheduling is utilized to differentiate the extent of these adverse effects.^4^ Since the introduction of the CSA in 1970, there has been limited published research to offer insights into the impact and usefulness of the numerical system on prescribing habits.^1^ Existing concerns that the scheduling system can negatively affect the practice of medicine have worsened over the past decade as a result of the opioid epidemic beginning in the early 2000s.^7^

While the CSA is useful in its original objective to limit “the illegal importation, manufacture, distribution, possession, and improper use of illicit substances”,^4^ categorized as CI, it is still unclear whether or not the current controlled substance scheduling system is capable of assisting prescribers to make informed decisions for controlled substances deemed to have “a useful and legitimate medical purpose and are necessary to maintain the health and general welfare of the American people” (CII – CV).^4^ This potential gap coupled with social factors centered around the misuse of illicit CI substances and opioids (CII) may have a significant impact on providing optimal patient care practices.^7^ A driver of this impact on patient care could be hesitancy to prescribe controlled substances due to potential for abuse and decreased patient adherence due to a medication being listed as a controlled substance.^8^, ^9^, ^10^, ^11^

Despite these issues with the scheduling system, there is clearly a necessity for regulation regarding medications that display potential for abuse or misuse. In the US, it is estimated that 21.4 % of people over the age of 12 have used illicit substances in the past year and another 44.5 % of people over the age of 12 have used prescription psychotherapeutic drugs, including pain relievers, tranquilizers, stimulants, and sedatives, in the past year.^12^^,^^13^ Furthermore, 5.8 % of individuals 12 years of age and older report misusing prescription psychotherapeutic medications in the past year.^14^ Given this fact, prescriber education, awareness, and understanding regarding the controlled substance scheduling system is an important factor in tackling all of these medico-legal issues to provide the most optimal patient care possible.

## 2 Materials and methods

### 2.1 Survey design

This was across-sectional design observation study using a mailed, anonymous, self-administered survey targeting physicians and nurse practitioners in four US states: Massachusetts, Rhode Island, Michigan, and Indiana. These four states were selected to align with the structure of this research's predecessor by Parker et al. which utilized two states in the Northeast and two states in the Midwest. The survey was designed to assess prescribers' knowledge and attitudes, as well as the impact of the controlled substance scheduling system on prescribing patterns.

This survey, 24 questions total, was designed to take between five and ten minutes to complete and was an electronic-based response system. The first 12 questions of the survey centered around demographic information. The second set of 12 questions were classified into three categories prior to distribution of the survey. Questions #1, #2, #3, and #4 were designed to assess prescribers background knowledge and understanding of the controlled substance scheduling system, Category 1. Category 2, consisting of Questions #5, #6, #7, and #8, were utilized to evaluate prescribers' attitudes towards the controlled substance scheduling system for topics related to patient adherence and personal training. Lastly, Questions #9, #10, #11, and #12 were intended to determine the impact the controlled substance scheduling system has on providing optimal patient care and physicians' willingness to prescribe medications.

The questions of the survey were developed to closely align to its predecessor from the 2009 study by Parker et al. The demographic questions (Part 1, Questions 1–12) were adjusted to assess which respondents self-identified as specialists and the percentage of patients typically seen for a psychiatric condition (Questions 2, 3, and 11). A specialist is defined as a MD/DO or NP with additional training in their specified therapeutic area (i.e., psychiatrist, cardiologist, etc.). The assessment questions (Part 2, Questions 1–12) were updated to include the provision in the CSA to allow for CII refills (Question 1), differentiate between state specific regulations (Question 4), and further assess potential impact of providing optimal patient care (Questions 6, 8, 9, and 10). The updated survey was validated via pilot testing and feedback from health care providers that would not be included in the study.

### 2.2 Subject selection and distribution

Surveys were distributed to a randomly selected sample of 100 physicians and 100 nurse practitioners per state in each of the four states (Massachusetts, Rhode Island, Michigan, and Indiana), totaling 800 subjects. By selecting 400 physicians for this study we kept to the same sample size as the original 2009 research. The 800 prescribers were identified utilizing the state licensing database and were selected from the database via a randomizer.

The electronic based survey was mailed to prescribers as a Quick Response (QR) code and Uniform Resource Locator (URL) as part of a cover letter encouraging participation and explaining the purpose of the research. Upon receipt of the mailed cover letter, prescribers could either scan the QR code to access the survey or manually enter the URL into a web browser. If contact information was available as part of each state's licensing database, an email reminder was also distributed on a weekly basis for the duration of the return period of one month. There were no incentives for participation in this study. The Purdue University Institutional Review Board reviewed and approved the procedures for this study (Reference #: IRB-2021-1889, received 19 Jan 2022).

### 2.3 Data entry and statistical analysis

The survey responses were automatically generated in and compiled through Qualtrics, then transferred into SAS for analysis.

For each dependent variable defined in Table 2, the frequency of response was tabulated and fitted with all the demographic independent variables in Table 1. Following screening, each identified important factor was then further tested utilizing either a Fisher's Exact Test for binary values or an Exact Pearson Chi-Square Test for dependent variables with multiple response categories. Analysis was performed using the SAS® Statistical Analysis program version 9.4 (SAS Institute, Cary, North Carolina).

**Table 1:** Respondent demographic breakdown.

Demographic Variable	Options	Respondents # (%)	Total respondents
1. MD/DO or NP	MD/DO NP	34 (45.95 %) 40 (54.05 %)	74
2. Specialist or Primary Care Provider	Specialist PCP	43 (58.11 %) 31 (41.89 %)	74
3. What is your specialty⁎	*N/A*⁎	*See supplementary table*	70
4. Which state do you primarily practice in	MA RI MI IN	12 (16.22 %) 11 (14.86 %) 38 (51.35 %) 13 (17.57 %)	74
5. Gender	Male Female Other/Prefer not to say	30 (40.54 %) 43 (58.11 %) 1 (1.35 %)	74
6. Years since completed first residency⁎	*N/A*⁎	*See supplementary table*	68
7. Years practicing specialty⁎	*N/A*⁎	*See supplementary table*	73
8. Practice setting (single, group, institution)	Single Group Institution	14 (19.18 %) 30 (41.10 %) 29 (39.73 %)	73
9. Practice setting (rural, urban, suburban)	Rural Urban Suburban	14 (18.92 %) 20 (27.03 %) 40 (54.05 %)	74
10. Typical # of patients seen per day⁎	*N/A*⁎	*See supplementary table*	73
11. Typical % of patients seen for a psychiatric condition⁎	*N/A*⁎	*See supplementary table*	72
12. DEA License	Yes No	67 (90.54 %) 7 (9.46 %)	74

MD = Doctor of Medicine; DO = Doctor of Osteopathic Medicine; NP = Nurse Practitioner; N/A = Not Applicable; PCP = Primary Care Provider; MA = Massachusetts; RI = Rhode Island; MI = Michigan; IN = Indiana; DEA = Drug Enforcement Administration.

⁎ = free response – see supplementary table.

Significance was defined as a *p*-value of 0.05 or less.

## 3 Results

### 3.1 Survey part 1 – Demographics

The demographic characteristics of respondents are summarized in Table 1, with the frequency of each response reported as raw numbers and as a percentage of total respondents. For demographic characteristics that were open responses a complete list of responses is provided in the supplementary section of this article.

### 3.2 Survey part 2 – Assessment

Table 2 summarizes the results of the twelve questions, in which a majority of the responses for each question suggested an overall lack of understanding of the controlled substance scheduling system (Questions 1 through 4), negative impact on patient care (Questions 5 through 8), or strong dissatisfaction with the impact the controlled substance scheduling system has with regards to providing patient care (Questions 9 through 12). Each question was further analyzed to determine the differences in responses across demographic groups.

**Table 2:** Survey results by question.

Question	Responses
1. Are you aware of the provision in the Controlled Substance Act that allows you to provide patients with multiple prescriptions at a time for CII medications?	Yes – 32 (47.06 %) No – 36 (52.94 %)
2. Are you aware that a drug could have no potential for physical or psychological dependence, but still be scheduled as a controlled substance on the basis of third party abuse?	Yes – 54 (78.26 %) No – 15 (21.74 %)
3. Are you aware that there are no federal limits on the number of refills for a CV medication?	Yes – 19 (27.94 %) No – 49 (72.06 %)
4. Are you aware of whether or not your state law has limitations on refilling CV medications?	Yes, aware of restrictions – 23 (34.33 %) Yes, aware there are no restrictions – 4 (5.97 %) No, not aware of any restrictions – 40 (59.70 %)
5. Do you believe your training (academic, experiential, etc.) provided adequate training regarding describing and detailing the controlled substance scheduling system?	Yes – 23 (33.82 %) No – 45 (66.18 %)
6. Do you believe insurance formularies are a barrier to patients who otherwise should be receiving a controlled substance?	Yes – 41 (61.19 %) No – 26 (38.81 %)
7. In your opinion, does the numbering system (I, II, III, IV, V) help or should medications be designated as “controlled” without ranking?	Yes, helps – 37 (55.22 %) No – 30 (44.78 %)
8. Do you believe restrictions on prescribing and refilling controlled substances has a negative impact on patient adherence for patients being maintained on a controlled substance medication?	Yes – 28 (41.18 %) No – 40 (58.82 %)
9. Do you believe the social stigma of CI and CII substances, as a result of the opioid epidemic, has negatively impacted patient care for patients needing CIII – CV medications?	Yes – 49 (72.06 %) No – 19 (27.94 %)
10. If a patient is chronically maintained on a controlled substance medication, for an indication other than pain, do you believe the scheduling of the medication as a controlled substance impacts your willingness to prescribe the medication compared to non-controlled substances? (i.e., anti-epileptic drugs in schedule IV or V compared to non-controlled)	Yes – 36 (52.94 %) No – 32 (47.06 %)
11. Overall, to what extent do you believe the scheduling system for controlled substances adequately conveys to physicians the information you need to assist you in making a prescribing decision?	1 – Not at all – 14 (20.59 %) 2 – Somewhat inadequate – 21 (30.88 %) 3 – Neither adequate or inadequate – 19 (27.94 %) 4 – Somewhat adequate – 13 (19.12 %) 5 – Excellent job – 1 (1.47 %)
12. Overall, what impact do you believe the scheduling system currently in place has on your ability to practice for patients who need medications that are scheduled?	1 – Strong negative – 13 (19.12 %) 2 – Minor nuisance – 26 (38.24 %) 3 – No impact – 20 (29.41 %) 4 – Helpful – 9 (13.24 %) 5 – Strong positive – 0 (0.0 %)

CI – CV = Controlled Substance Schedule 1 through 5;

## 4 Discussion

### 4.1 Prescriber baseline knowledge

The first four questions were designed to evaluate the background knowledge of respondents regarding the CSA. An update in 2007 to the CSA, enables healthcare providers to write up to a 3-month supply of a CII medication via three separate prescriptions with appropriate “do not fill before” dates.^15^ Previously, there were no such exemptions for CII medications, so it is understandable why a slight majority of respondents were unaware of this regulation. The only statistically significant difference across demographic groups for this response was between male and female respondents (75.86 % vs 35.90 %, *p*-value 0.0014). Another difference trending towards but not statistically significant was MD/DO respondents were less likely to respond correctly compared to nurse practitioners (33.33 % vs 57.89 %, *p*-value 0.0535).

Another aspect the CSA specifies is the placement of a controlled substance solely based on the potential for third-party abuse and misuse.^4^ To assess prescribers' knowledge with regards to this aspect Question #2 was implemented. Most respondents indicated that they were aware of this provision to the CSA (54 of 69, 78.26 %). Although responses across all demographics correctly indicated they were aware of this provision, female respondents were significantly more likely to respond correctly (87.50 % vs 65.52 %, *p*-value 0.0397) when compared to male respondents; and similar to Question #1, nurse practitioners were more likely to respond correctly compared to physicians (84.21 % compared to 70.97 %), although this also was not statistically significant (p-value 0.2441).

The CSA restricts the quantity of refills and duration of validity a prescription may have based on the medication's schedule. For CII substances, a singular prescription may not have any refills and is only valid for 30 days from the date written. Whereas CIII and IV are limited to five refills of one month supply and are valid for a six-month period. The CSA does not specify any additional restrictions or limitations for CV controlled substances compared to non-controlled medications.^16^ However, several states, including Massachusetts and Rhode Island, have state-specific regulations to implement restrictions akin to that of CIII and CIV substances.^17^^,^^18^ While other states, including Michigan and Indiana, do not have additional state-specific regulations regarding CV substances. Questions #3 and #4 were used to assess if the prescribers were aware of this lack of federal regulation and the existing state-specific requirements. Surprisingly, a majority of respondents were unaware of this nuance in the CSA (49 of 68, 72.06 %), as well as whether or not their state of primary practice had additional state-specific restrictions (40 of 67, 59.70 %). These responses were consistent across all demographic groups for Question #3, but when comparing physicians to nurse practitioners there was a statistically significant difference (*p*-value 0.0010) indicating that physicians were less aware of their state-specific regulations (83.33 % vs 40.54 %). Furthermore, when comparing responses to current state regulations, 60 % percent of prescribers from Massachusetts (*n* = 10), 18.2 % of prescribers from Rhode Island (*n* = 11), 16.7 % of prescribers from Indiana (*n* = 12), and zero prescribers from MI (*n* = 34) attested that they were aware and accurately answered Question #4.

The responses to this first set of questions demonstrate an overall lack of understanding of the CSA and federal controlled substance regulations, which is consistent with the findings from the 2009 study by Parker, et al.,^1^ Interestingly, as shown in Question #4, these results are also indicative that prescribers are generally unaware of regulations in their state of primary practice. However, differences among demographic groups highlight the importance of mid-level practitioners, such as nurse practitioners, on providing routine patient care, as this demographic subgroup answered the first grouping of questions more accurately than physicians. Given the increased emphasis on controlled substances due to the opioid epidemic, these results demonstrating an overall lack of understanding of controlled substance regulations aligning with results from 2009 is concerning and highlights a need for greater prescriber education regarding medicolegal issues.

### 4.2 Prescriber attitudes

The next set of four questions (#5 through #8) were used to determine prescribers' attitudes regarding the utility of the controlled substance scheduling system. Question #5 asked whether or not prescribers felt they have received adequate training through academic schoolings and post-graduate experiential training regarding the controlled substance scheduling system. A majority of respondents (45 of 68, 66.18 %) designated they felt they did not receive adequate training. This response was consistent across all demographic groups and indicates a correlation between lack of education regarding the controlled substance scheduling system with the overall lack of understanding demonstrated in Questions #1 through #4.

Questions #6 and #8 were utilized to assess prescribers' perceived impact the controlled substance scheduling system has on patient adherence, either directly or indirectly through insurance formularies. For Question #6, prescribers were asked if they felt insurance formularies acted as a barrier to patients who otherwise should be receiving controlled substances. Most respondents confirmed this is the case (41 of 67, 61.19 %). Although not statistically significant, there were some differences between demographic groups for Question #6, including specialist compared to primary care providers (65.00 % vs 55.56 %, *p*-value 0.4560), practice setting – single compared to group compared to institution (53.85 % vs 68.97 % vs 56.00 %, *p*-value 0.5739), and geographic setting – rural compared to urban compared to suburban (57.14 % vs 50.00 % vs 68.57 %, *p*-value 0.3782). While a majority of prescribers did note that insurance formularies act as a barrier to patients in need of controlled substance medications, over half did not believe that restrictions centered around prescribing and refilling controlled substances impacted patient adherence (40 of 68, 58.82 %) in Question #8.

However, for Question #8, there were statistically significant differences in responses between specialists compared to primary care providers (52.50 % vs 25.00 % indicating yes, *p*-value 0.0272) and geographic setting – rural compared to urban compared to suburban (7.14 % vs 50.00 % vs 50.00 % indicating yes, p-value 0.0123). Other demographic differences for Question #8 that were not statistically significant included state of practice – MA, RI, MI, or IN (50.00 % vs 45.45 % vs 45.71 % vs 16.67 % indicating yes, *p*-value 0.3152) and practice setting – single compared to group compared to institution (38.46 % vs 27.59 % vs 56.00 % indicating yes, *p*-value 0.1181).

Lastly, Question #7 was intended to assess if differentiation in ranking of controlled substances was beneficial or if the term “controlled” should be a blanket statement for all controlled substances. With regards to this, a majority of prescribers (37 of 67, 55.22 %) preferring a ranking system. Differences across demographics for Question #7 included specialist compared to primary care provider (50.00 % vs 62.96 %, p-value 0.3270), practice setting – single compared to group compared to institution (61.54 % vs 64.29 % vs 44.00 %, p-value 0.3421), and geographic setting – rural compared to urban compared to suburban (69.23 % vs 44.44 % vs 55.56 %, p-value 0.4320), but none were statistically significant.

Similar to the first grouping of questions, these results for the second grouping match the findings from the 2009 study by Parker, et al. demonstrating an overall negative attitude towards the controlled substance regulations and their impact on providing optimal care.^1^ Questions #6 and #8 were de novo questions that additionally highlighted the effects of the controlled substance scheduling system on prescriber attitudes via insurance formularies and refill restrictions. Differences in response frequency among demographic groups existed for the second grouping of questions as well. Most notably, prescribers identifying as specialists tend to answer more negatively compared to primary care providers, as well as prescribers in urban settings when compared to prescribers in rural settings.

Despite the overall indication of a negative attitude towards the controlled substance scheduling system, it should be noted that the results for Questions #7 and #8 demonstrate the benefit of the controlled substance scheduling system in achieving its original objective of protecting public health.^4^ The responses to this second group of questions further demonstrates the clear necessity for a scheduling system to protect the public health, while simultaneously acknowledging that improvements must be made to avoid hindering optimal patient care.

### 4.3 Impact of scheduling

The final set of questions (Questions #9 through #12) evaluated the perceived impact on providing optimal care for patients in need of controlled substance medications. Question #9 was intended to assess the impact of the social stigma of CI and CII prescriptions, as a result of the opioid epidemic, on prescribing patterns for CIII – CV medications. As mentioned previously, illicit substances, classified as CI, and opioids, designated as CII medications, have an ever-increasing social stigma regarding their use. Regarding the impact of this social stigma, prescribers strongly felt a negative impact on patient care for patients in need of CIII through CV medications (49 of 68, 72.06 %). As mentioned previously, the opioid epidemic has caused an increased emphasis on appropriate stewardship of controlled substances, particularly over the past decade since Parker, et al., which was reflected in the responses to this question. There were no differences across demographic groups for Question #9.

Elaborating on Question #9 regarding impact on providing optimal medical care for patients in need of non-narcotic controlled substances, prescribers were then asked about their willingness to prescribe controlled substances as maintenance therapy for patients, for indications other than pain, in Question #10. Although the vast majority of respondents suggested the social stigma surrounding CI and CII medications negatively impacts care for patients receiving CIII through CV medications, a small majority of respondents (36 of 68, 52.94 %) stated that a medication being designated as a controlled substance affects their willingness to utilize it for chronic therapy. Slight differences in responses existed across demographics include specialist vs primary care provider (62.50 % vs 39.29 %, *p*-value 0.0844), practice setting – single compared to group compared to institution (61.54 % vs 44.83 % vs 56.00 %, p-value 0.5957), and geographic setting – rural compared to urban compared to suburban (50.00 % vs 61.11 % vs 50.00 %, p-value 0.8043), but none were statistically significant.

The utility of the current scheduling system in relaying information regarding the potential for abuse of controlled substances and the direct impact the controlled substance scheduling system has on providing optimal patient care were evaluated using Questions #11 and #12, respectively. With regards to the ability of the scheduling system to adequately convey information needed to make an informed prescribing decision, a small majority of respondents (35 of 68, 51.47 %) suggested the system is inadequate while approximately one fifth of respondents (14 of 68, 20.59 %) held a favorable view of the system. When further analyzed based on demographic data, physicians were more likely to respond negatively to Question #11 compared to nurse practitioners (*p*-value 0.0012). These results were similar for Question #12 regarding the direct impact of the controlled substance scheduling system on providing optimal patient care, with a majority of respondents (39 of 68, 57.36 %) indicating a detrimental impact and only nine respondents (13.24 %) indicating the scheduling system is beneficial. When prescribers' responses were compared by their state of primary practice – Massachusetts, Rhode Island, Michigan, or Indiana – a statistically significant difference was seen indicating prescribers from Michigan are more likely to respond favorably (p-value 0.0088) compared to other respondents.

As with the other groupings, the results for the final group of questions designated to evaluate the impact of the scheduling system on providing optimal patient care line up with previous findings from Parker, et al..^1^ Similarly, the responses to Questions #9 through #12 are indicative of a perceived negative impact on patient care caused by the controlled substance scheduling system. Questions #9 and #10 were de novo in this survey and highlight the influence of the social stigma surrounding controlled substances, because of the opioid epidemic, with results demonstrating this negative impact on providing optimal patient care for patients in need of medications designated as CIII - CV. While Questions #11 and #12 were re-used from the 2009 study by Parker, et al., with results indicating prescribers' negative perception of the utility of the current scheduling system. As previously mentioned, there is a clear need for such a system to protect public health from medications that exhibit the potential for abuse, however, the current system enacted by the CSA appears to be detrimental to the usual course of medical practice. The results of this survey confirm this affirmation by Parker, et al., and further display the need for investigation into how to improve the scheduling system.^1^

### 4.4 Study strengths and limitations

Eight hundred surveys were sent to randomly selected physicians in four states, Massachusetts, Rhode Island, Michigan, and Indiana in January 2022 and responses were accepted until March 2022. The electronic-based survey was distributed via physical mail with a QR code in an attempt to optimize response rate.^19^ The utilization of the electronic-based survey enabling ease of completion and back-end compilation of preliminary results was a key strength of this study. An additional strength of this work is the commonality of questions between this survey and its predecessor, Parker et al.,^1^ which allowed for direct comparison on how prescribers' attitudes have changed over the course of the past decade regarding controlled substances and the scheduling process.

Recent reviews and analyses have noted that the response rate for prescriber surveys has been gradually decreasing over the past few decades, with some noting an average response rate around 10 % to 15 % for electronic based surveys.^19^^,^^20^ Due to these factors, a minimum response rate for this research was determined a priori to be 10 %. Additionally, barriers for this survey research included lack of incentive for completion,^19^^,^^21^ inaccessibility of public information in Massachusetts and Indiana, and excessive healthcare provider fatigue due to the COVID-19 pandemic. The licensing database for Massachusetts and Indiana do not provide email addresses for follow up notifications, and the database for Indiana does not provide updated mailing addresses. It is possible that these factors could have negatively impacted the overall response rate. Nonresponse bias could be a potential limitation of this study. However, a 10 % response level was considered an adequate sample to draw conclusions compared to the study's predecessor.

An additional limitation of this study includes overall generalizability due to surveying 4 of the 50 states. However, given that the overall trends and results of the study were consistent across all respondents, the findings of this study can be adequately generalized as representative of the entire target population. Additionally, the primary aim of this study was to compare this research's findings with that of Parker et al., so four states were chosen for consistency.

Despite these limitations, prescribers across all demographic groups provided consistent responses across the three categories of prescriber knowledge, attitudes, and impact, suggesting an overall negative attitude towards the controlled substance scheduling system, deficiencies in understanding of specific regulations within the CSA or state law, and a negative impact on providing optimal patient care.

## 5 Conclusion

This research was performed to follow up on the 2009 study “Physicians' knowledge and attitudes toward scheduling” by Parker, et al., to assess the level of general knowledge and attitudes of prescribers regarding the controlled substance scheduling system, as well as the impact of the controlled substance scheduling system on prescribing habits and how these insights may have changed over the past decade. While the current survey was updated from its predecessor, many of the questions were identical or substantially similar.

Given the increased consideration placed on opioid stewardship, as a direct result of over 1 million opioid overdose deaths in the United States since 2000, it is not surprising that the results of this survey line up with the findings from the 2009 study. Overall, there was consistency across demographics expressing a lack of understanding about, negative attitude towards, and detrimental effects on medical practice of the controlled substance scheduling system.

Notably, there were three questions that stood out as having shown a level of change – prescriber awareness regarding multiple CII prescriptions, value of the numerical system (vs just controlled) to scheduling and belief the prescribers' training was adequate regarding controlled substances. For awareness of multiple CII prescriptions, there was a marked improvement in awareness which may reflect the regulations having been established for a longer period. Regarding the value of the numerical system, there was a decrease. This could reflect either a disagreement regarding where items are placed or that the value is in creating the recognition of potential issues in a dichotomous manner rather than having the scale. Finally, regarding training the respondents cast further concern regarding the amount of training they receive for scheduled products indicating the issue is potentially getting worse and not better.

Although the CSA is effective in its original intent of protecting the American public from the dangers of illicit substances, these results indicate that the CSA and the controlled substance scheduling system may serve as a hindrance to providing optimal patient care for patients in need of medications designated as controlled substances. The results of this survey confirm the findings of Parker, et al., and further display the need for investigation into how to improve the controlled substance scheduling system in the United States.

## Funding

This research did not receive any specific grant from funding agencies in the public, commercial, or not-for-profit sectors.

## Credit authorship contribution statement

**Michael R. Barnes:** Writing – review & editing, Writing – original draft, Validation, Project administration, Methodology, Investigation, Formal analysis, Data curation, Conceptualization. **Yijia Luo:** Writing – review & editing, Supervision, Project administration, Methodology, Conceptualization. **Jonathon M. Parker:** Writing – review & editing, Supervision, Methodology, Conceptualization. **Brian M. Shepler:** Writing – review & editing, Supervision, Project administration.

## Declaration of competing interest

The authors do not have any conflicts of interest to disclose related to this research.

## Appendix A Supplementary data

Supplementary material Responder demographics Supplementary material
