# Supplementary material for: Prescribers' perspectives: The impact of the controlled substance scheduling system on providing optimal patient care
Source: Explor Res Clin Soc Pharm. 2024 Sep 21;16:100511. doi: 10.1016/j.rcsop.2024.100511 (PMC11474174; doi:10.1016/j.rcsop.2024.100511)
Supplement: Supplementary material — Responder demographics [file mmc1.docx]

**Supplementary Table**

| **Complete Response List – Open Response Demographic Questions** | |
| --- | --- |
| Question 3: What is your specialty? (Please write “N/A” if not applicable) | Acute Care (1)  Acute Care Gerontology (1)  AGACNP Gerontology (1)  Anesthesiology (1)  Cardiology (4)  College Health (1)  Dermatology (1)  Emergency Medicine (2)  Family Medicine/Practice (8)  General Surgery (1)  Gerontology (1)  Hematology/Oncology (1)  Hospice and Palliative Care (1)  Infectious Disease (1)  Internal Medicine (5)  Maternal/Fetal Medicine (2)  Neurology (1)  Nephrology (2)  OB/GYN (6)  Occupational Medicine (3)  Oncology (1)  Ophthalmology (1)  Orthopedic (2)  Pain Management (1)  Pathology (3)  Pediatrics (4)  Psychiatry (2)  PMR (1)  Rehabilitation (1)  Urgent Care (1) |
| Question 6: How many years has it been since you completed your first residency? | One (5)  Two (5)  Three (5)  Four (1)  Five (1)  Six (2)  Seven (1)  Nine (4)  Ten (4)  Eleven (1)  Twelve (1)  Fourteen (1)  Fifteen (1)  Sixteen (1)  Nineteen (1)  Twenty (4)  Twenty-one (1)  Twenty-two (3)  Twenty-three (1)  Twenty-four (1)  Twenty-five (1)  Twenty-six (1)  Twenty-seven (1)  Twenty-eight (1)  Thirty (1)  Thirty-one (1)  Thirty-five (2)  Thirty-six (2)  Thirty-seven (1)  Thirty-nine (1)  Forty (1)  Forty-three (1)  Forty-four (1)  Forty-five (1)  Forty-six (1)  Forty-eight (1)  Fifty (1) |
| Question 7: How many years have you been practicing in your specialty? (Please write “N/A” if not applicable) | Less than One (2)  One (10)  Two (1)  Three (4)  Four (3)  Five (1)  Six (1)  Seven (2)  Eight (1)  Nine (3)  Ten (4)  Eleven (1)  Thirteen (2)  Fourteen (1)  Sixteen (3)  Nineteen (2)  Twenty (2)  Twenty-one (1)  Twenty-two (1)  Twenty-four (2)  Twenty-five (3)  Thirty (3)  Thirty-one (1)  Thirty-two (1)  Thirty-three (1)  Thirty-four (1)  Thirty-five (1)  Thirty-seven (1)  Thirty-eight (1)  Forty (1)  Forty-two (1)  Forty-four (1)  Forty-five (1)  Forty-eight (1)  Fifty (1) |
| Question 10: What is the typical number of patients you see per day? | Zero (1)  One (2)  Three (2)  Four (1)  Five (1)  Six (2)  Six to Eight (1)  Eight (3)  Eight to Twelve (1)  Ten (4)  Ten to Twenty (1)  Twelve (4)  Thirteen (5)  Fourteen (2)  Fifteen (5)  Fifteen to Twenty (1)  Fifteen to Thirty (1)  Sixteen (2)  Seventeen (3)  Eighteen (4)  Twenty (12)  Twenty to Thirty (1)  Twenty-two to Twenty-six (1)  Twenty-five (3)  Twenty-five to Fifty (1)  Thirty (1)  Forty (2)  Fifty (3) |
| Question 11: What is the typical percentage of patients you see per day for a psychiatric condition (i.e., anxiety, depression, etc.)? | Zero (23)  One (2)  One to Three (1)  Two (2)  Four (2)  Five (4)  Six (1)  Eight to Ten (1)  Ten (4)  Fifteen (3)  Twenty (5)  Twenty-five (6)  Thirty (5)  Fifty (1)  Sixty (2)  Sixty-six (1)  Seventy (2)  Seventy-five (2)  One Hundred (3) |
